# Assessing patient satisfaction with a microsuction service in general practice: a comparative study

**DOI:** 10.3399/bjgpopen19X101649

**Published:** 2019-06-12

**Authors:** Ruairi Hasson, Eoin McDermott, Karena Hanley, Camilla Carroll, Claire Collins

**Affiliations:** 1 GP Registrar, Irish College of General Practitioners, Dublin, Ireland; 2 GP Registrar, Irish College of General Practitioners, Dublin, Ireland; 3 National Director of GP Training, Irish College of General Practitioners, Dublin, Ireland; 4 National Lead for ENT Education in Primary Care, Royal College of Surgeons Ireland, Dublin, Ireland; 5 Director of Research & Innovation, Irish College of General Practitioners, Dublin, Ireland

**Keywords:** patient satisfaction, general practice, otolaryngology, cerumen, otitis externa, delivery of health care, integrated

## Abstract

**Background:**

In the UK, about 2.3 million people each year require intervention for wax impaction, while otitis externa accounts for just over 1% of general practice consultations. Aural microsuction of debris from the ear canal is a commonly performed procedure within the ear, nose, and throat (ENT) outpatient clinic. This article examines the patient acceptability of an aural microsuction service delivered in general practice.

**Aim:**

To determine patient satisfaction following the introduction of a new microsuction service in general practice compared with a hospital-delivered service.

**Design & setting:**

This is a prospective comparative study in two rural general practices in Ireland and the emergency department (ED) of the Royal Victoria Eye and Ear Hospital (RVEEH), Dublin.

**Method:**

A 3-month period of data collection on usual care of 56 patients in general practice was followed by a 3-month period of GP-intervention data collection on 67 patients. Comparative data were collected on 37 patients who attended the RVEEH for the same intervention procedure. Patients completed a validated patient satisfaction questionnaire (PSQ-18).

**Results:**

Both general practice groups scored significantly higher in all seven aspects of medical care than the RVEEH cohort. Patients in the GP-intervention group scored significantly higher in terms of satisfaction with procedure technique compared with the usual care GP group.

**Conclusion:**

The provision of microsuction as a service in general practice confers as much or more patient satisfaction as the provision of the service in a hospital setting.

## How this fits in

There are multiple studies examining the impact on patient satisfaction of the introduction of specialties into primary care that historically were found only in secondary care.^1–4^ This study shows that a microsuction service in the general practice setting confers patient satisfaction, delivering care in a setting that is preferable to patients. This is consistent with much of the international research supporting a restructuring of healthcare provision towards integrated care and away from a hospital-centric model.^5,6^

## Introduction

In the UK, about 2.3 million people each year require intervention for wax impaction.^7^ Otitis externa accounts for just over 1% of all general practice consultations,^8^ with 3% of cases seen referred to secondary care.^9^ Aural microsuction of debris from the ear canal is a commonly performed procedure within the ENT outpatient clinic.^10^ Cashman *et al* ^11^ found that over half of all otology referrals were for otitis externa and wax impaction, conditions that may require microsuctioning, which is not routinely provided by GPs in Ireland. Microsuction has the advantage that it is often quicker, allows direct visualisation and does not expose the ear to moisture.^12^ Also, studies have shown that otitis externa is overly treated with antimicrobials in the community,^13^ driven by a lack of community access to microsuction facilities.

The 2018 National Institute for Health and Care Excellence (NICE) guidelines have already supported the use of microsuction in primary care for aural wax removal.^7^ By confirming the patient acceptability of the service, this article makes a stronger case for provision of funding for training and equipment to facilitate microsuction in the community. Several studies have examined patient satisfaction, which is consistently higher, following the introduction of services to primary care that historically are found in a secondary care setting.^1–4^ The aim of this study is to determine whether provision of microsuction in general practice leads to at least as much patient satisfaction as provision of this service in the hospital setting.

## Method

This was a prospective comparative study, comparing a new microsuction service in primary care, in terms of patient satisfaction, with the current secondary care service through use of a validated Patient Reported Experience Measure (PREM) questionnaire: the PSQ-18.^14^ It was conducted in two rural general practices in Ireland and the ED of the RVEEH. Data were collected on usual care for the first 3 months of 2018 (until 31 March 2018) followed by a 3-month period of intervention data collection (until 30 June 2018) in two GP practices. Data were collected on patients who underwent microsuction for the second 3-month period (until 30 June 2018) in the RVEEH. At midday, Monday to Friday, reception staff gave the questionnaire to the next two ENT patients. Midday was chosen as this was felt to best represent the average waiting time for patients. This served as a comparative sample of patients attending the RVEEH secondary care service.

Prior to commencement, using PS Software sample size calculation and taking a 95% confidence interval (CI) and an 80% power to detect differences, 36 patients were required to detect a difference in mean overall satisfaction scores of 28 compared with 30 between the groups. Owing to lower numbers than expected in the RVEEH sample, the sample size in the end was primarily a convenience sample, taking into account practical considerations.

Inclusion criteria were any patient aged >18 years, presenting with earwax impaction or otitis externa. Excluded were individuals with a language barrier, intellectual disability, or other capacity issues.

During the initial pre-intervention 3-month period, any patients identified with wax impaction or otitis externa were offered usual care and asked to complete a questionnaire. This was to be the intra-practice control. It was planned to offer all eligible patients microsuction during the intervention periods. In the course of busy practice, a small number of patients were not diverted to the research leads and were instead treated by other members of the practice team without the offer of microsuction. Of those offered usual care or microsuction by the researchers, there was a unanimous uptake for the new service. It was not expected that the patients who were not offered microsuction during the intervention period differed appreciably from those who availed of microsuction, but there is no available data on this.

The microsuction service was provided by two final-year general practice trainees, supervised by their trainers. Both trainees received RVEEH ENT consultant-led proficiency training in microsuction. The RVEEH was also involved in remote supervision of the newly introduced microsuction service and professional indemnity for this pilot was agreed with insurers.

Data for patient satisfaction were collected using the PSQ-18 questionnaire,^14^ a validated PREM. This measures the patient satisfaction according to a Likert scale in seven separate areas, as denoted in Box 1. For each area, a scale of 1–5 is presented, with a higher score denoting an increased level of satisfaction. All completed questionnaires were returned in an anonymous envelope into a locked box. Data were entered onto a spreadsheet in Excel and then transferred to SPSS (version 24) for non-parametric analysis. Independent samples Kruskal-Wallis tests were used to test for differences in satisfaction rates between the three groups.

Box 1. **PSQ-18 areas of assessment**

**Table:** 

**Areas of assessment**
General satisfaction
Technical quality
Interpersonal manner
Communication
Financial aspects
Time spent with doctor
Accessibility and convenience

Further information on the PSQ-18 questionnaire and instructions for scoring is available from the author on request.

## Results

**Figure 1.**
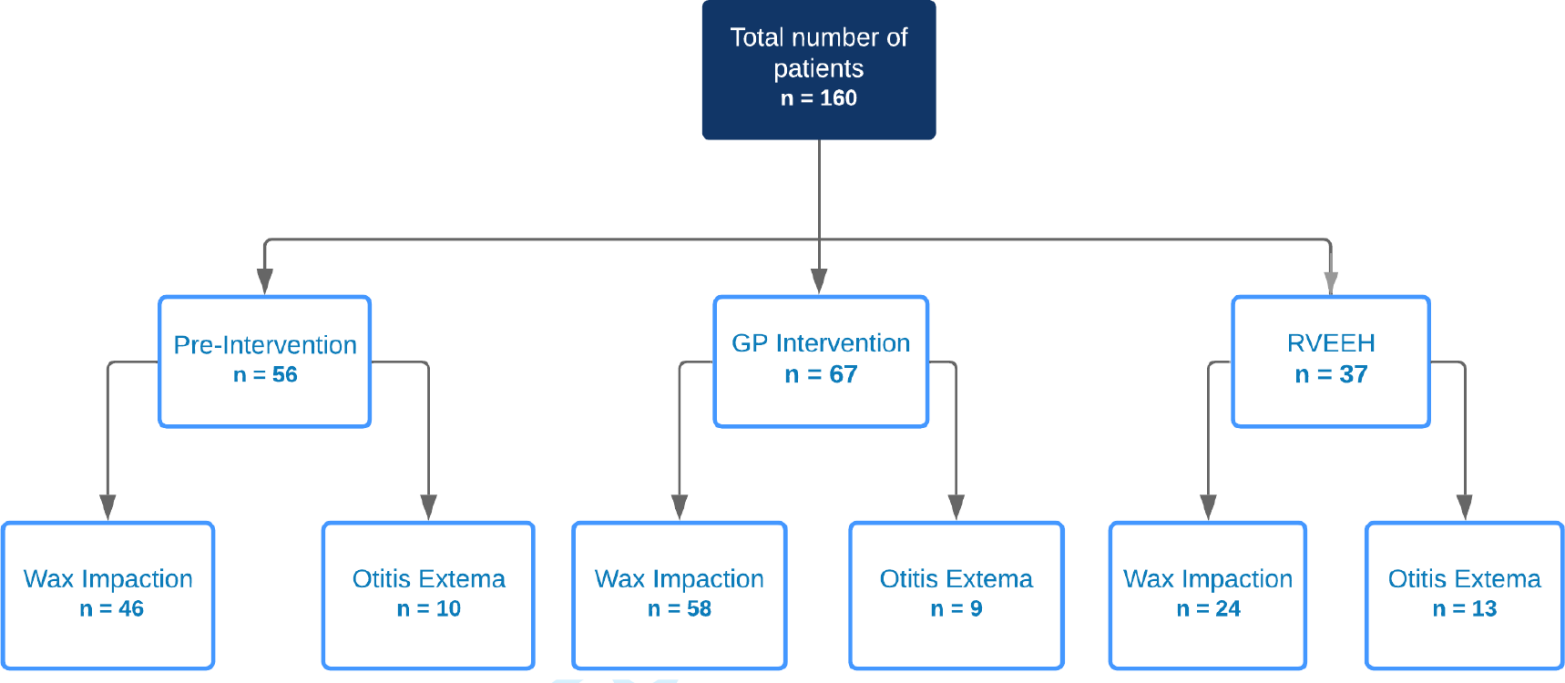
. Number of patients in each of the groups (pre-intervention, intervention, and control)

A total of 160 patients were invited to participate in the study. The breakdown of patients for each group is shown in Figure 1. For the GP intervention group, the response rate to the invitation of the study was 100% positive. In both GP groups, the response rate of questionnaires returned during the 6-month period was 100%.

On the median Likert score, higher satisfaction occurred in the GP-intervention group compared with the same service provided in the hospital group across all areas assessed by the PSQ-18. Mean scores for 'satisfaction' with the service were 4.40 in the GP-intervention group in comparison with 3.96 for the RVEEH control (*P*<0.002). Scores for all seven aspects of the PSQ-18 are shown in Table 1. These results were independent of whether analysis was combined for wax impaction and otitis externa, or whether each were analysed separately.

**Table 1.** Average (mean) Likert scales in each of seven areas for the three groups, and Kruskal-Wallace test of significance

**Wax and otitis externa**	**Satisfaction**	**Technical quality**	**Interpersonal manner**	**Communication**	**Financial aspects**	**Time spent with doctor**	**Accessibility and convenience**
GP-intervention group	4.40	4.43	4.43	4.49	4.04	4.10	3.84
Pre-intervention group	4.35	4.10	4.30	4.37	3.75	4.16	3.64
RVEEH control	3.96	3.77	3.97	4.04	3.81	3.74	3.44
Kruskal-Wallace difference test of significance^a^	*P*<0.002	*P*<0.000	*P*<0.000	*P*<0.000	*P*<0.000	*P*<0.02	*P*<0.03

RVEEH = Royal Victoria Eye and Ear Hospital, Dublin.

a Between GP-intervention and RVEEH care.

There were no statistical differences found in satisfaction scores between the GP-intervention group and the pre-intervention group, with the exception of 'technical quality'. This showed a statistically significant higher satisfaction score (*P*<0.04) in the GP-intervention group.

Patients were also invited to make comments on the service they had received. Almost all the feedback in the GP-intervention group was positive:

- *'I think that the microsuction service to be available here in the practice is a great idea. This has saved me the hassle and inconvenience of having to drive to Dublin. Well done!'*
- *'Very comfortable and pain free.'*
- *'A wonderful experience, the doctor was a complete expert — fast, effective, and pain free.'*
- *'Definitely recommend it — so good not to have to go to the hospital for such a simple procedure*.'
- *'Strongly in favour of this, excellent for the practice to be able to offer this service. Thank you!'*
- *'Very efficient reassuring service. Many thanks!'*
- *'Very good, better than the water cannon.'*

Two negative responses in the GP-intervention group were recorded:

- *'GP microsuction should never substitute being seen by the ENT specialist plus procedure with regularity.'*
- *'I prefer to be seen by a specialist! Thank you!'*

## Discussion

### Summary

Satisfaction rates of aural microsuctioning, in particular the technical aspect, were generally higher in all aspects in the general practice setting in contrast to the hospital setting. All satisfaction scores for the GP-intervention were higher than the pre-intervention, but this was not a statistically significant difference. Perhaps this is because a lower number of cases collected than planned reduced the power of this study, but it may indicate a general preference among patients for care delivered in primary care, consistent with much of the international research.^1–4^ The technical aspect of aural microsuction, when performed in the general practice setting, produced statistically higher satisfaction rates than either usual general practice care or microsuction in the hospital setting. This study shows that aural microsuction results in at least equivalent patient satisfaction when delivered in a general practice setting as when delivered in a hospital setting.

### Strengths and limitations

The study design allowed for direct comparison of setting impact for the same procedure delivered in hospital or in the community. The study was conducted over a sufficiently long period that allowed for the service to get properly established. The PREM questionnaire used for data collection (PSQ-18) is an adaptable, reliable, and validated tool for use in various settings.^15^

It is possible that the failure to offer microsuction universally to all patients during the study period may mask an undetected bias. Most patients in general practice will never have had microsuction and may have been positively biased towards the new treatment. Another source of bias is that the GP-intervention service was delivered by the investigators. Patients may have been aware of this and have been reluctant to offend in their answers. Anonymity of responses helped to reduce this response bias.

### Comparison with existing literature

There are many studies examining the impact on patient satisfaction following the introduction of specialist services to primary care that historically are found only in a secondary care setting.^1–4^ These articles show that patient satisfaction is consistently higher when receiving a similar service in general practice as opposed to secondary care. The results of this study are consistent with these findings. While the format of the questionnaire did not allow for the extraction of exact reasons for the higher satisfaction scores, previous studies^1–4^ suggest it may be attributable to a number of factors, including accessibility, less travel time and costs, and less time off work. Patients may also feel more comfortable in their familiar local service in comparison with a large hospital setting.

### Implications for practice

The authors propose that providing a service such as microsuction in general practice, if properly funded, is consistent with the international shift away from a hospital-centric model and towards services delivered in primary care.^5,6^

NICE guidelines,^7^ along with the American Academy of Otolaryngology,^12^ recommend using either cerumenolytics, syringing, or microsuction based on adequate skills and training. While this study was one of patient acceptability, it encourages GPs to develop skills to provide this service.
